# Relationships between Serum Luteinizing Hormone Level, Endometrial Thickness and Body Mass Index in Polycystic Ovary Syndrome Patients with and without Endometrial Hyperplasia

**DOI:** 10.22074/ijfs.2016.4766

**Published:** 2016-04-05

**Authors:** Fariba Ramezanali, Gholamreza Khalili, Arezoo Arabipoor, Narges Bagheri Lankarani, Ashraf Moini

**Affiliations:** 1 Department of Endocrinology and Female Infertility, Reproductive Biomedicine Research Center, Royan Institute for Reproductive Biomedicine, ACECR, Tehran, Iran; 2 Department of Epidemiology and Reproductive Health, Reproductive Epidemiology Research Center, Royan Institute for Reproductive Biomedicine, ACECR, Tehran, Iran

**Keywords:** Polycystic Ovary Syndrome, Endometrial Hyperplasia, Luteinizing Hormone, Body Mass Index

## Abstract

**Background:**

The endometrial hyperplasia measured by ultrasound in polycystic ovary syndrome (PCOS) women is strongly related to pathologic endometrial thickness, but there is no consensus on the relation between serum luteinizing hormone (LH) and either of these factors: pathologic endometrial hyperplasia and body mass index (BMI).

**Materials and Methods:**

In this observational cross-sectional study, three hundred fifty infertile PCOS women were involved in this research. An endometrial biopsy was taken by using a pipelle instrument, regardless of menstrual cycle’s day and all samples were reported by the same pathologist. Basal serum LH level was compared between two subgroups (hyperplasia and non-hyperplasia). The intended population was divided into three groups according to BMI and basal serum LH, later on the comparison was made in three groups. Chi-square test was applied to compare nominal variables between groups. Mann-Whitney U, and one way ANOVA tests were used to compare means on the basis of the result of normality test.

**Results:**

The frequency of endometrial hyperplasia was 2.6%. Endometrial thickness in the patients with endometrial hyperplasia was significantly higher than that of a normal endometrium (10.78 ± 3.70 vs. 7.90 ± 2.86 respectively, P=0.020). There was no relation between endometrial hyperplasia and serum LH (P=0.600). The ANOVA test showed serum LH levels were not the same among three BMI groups (P=0.007). Post hoc test was also performed. It showed that the LH level in normal BMI group was significantly higher than those of other groups (P=0.005 and P=0.004), but there was no statistical difference between overweight and obese groups (P=0.8). We found no relationship between BMI and endometrial thickness in PCOS patients (P=0.6).

**Conclusion:**

Sonographic endometrial stripe thickness is predictive for endometrial hyperplasia in PCOS women. We could not find out any relationship between serum LH level and BMI with endometrial thickness in PCOS patients. However, our study confirmed a diverse relationship between serum LH level and BMI in PCOS patients.

## Introduction

Polycystic ovary syndrome (PCOS), the most common cause of anovulatory infertility, affects 5-10% of women of fertile age (1). The definition of PCOS in compliance with the 2003 Rotterdam criteria was confirmed in ESHRE/ASRM consensus meeting, provided that at least two out of three following features exist: oligo-ovulation or anovulation, elevated levels of androgens (Hyperandrogenemia) or clinical manifestations of androgen excess (Hyperandrogenemism) and polycystic ovaries as observed by ultrasonography (2). The endometrium in PCOS women has a wider spectrum compared to that of normal endometrium and has a higher incidence of hyperplasia and carcinoma (3, 4).

The incidence rate of hyperplasia in PCOS women is higher than that of normal women (5, 6). High prevalence of endometrial hyperplasia in such women is attributed to persistently high levels of estrogen (mainly estrone) without progesterone (that inhibit proliferation). However, the endometrial function of women with PCOS completely differs from a normal endometrium and is consistent with a predisposition to hyperplasia and carcinoma (7-10).

Because of the increased gonadotropin-releasing hormone (GnRH) pulsatility, luteinizing hormone (LH) hyper secretion is one of the hallmarks of PCOS. Increasing levels observed in about 70% of PCOS patients with elevated LH pulse amplitude and increased LH pulse frequency leading to a two to three fold elevation in serum LH level versus follicle stimulating hormone (FSH) serum level (11).

An increased LH/FSH has been used as a diagnostic test for PCOS for many years, but recent consensus recommendations are against the ones which were used before (12). Some studies reported the basal serum LH levels correlated inversely with body mass index (BMI) in PCOS patients (13, 14), but it is not approved by Hendriks et al. (6), who found no relationship between BMI and LH level in PCOS patients. The aim of this study is to investigate the relationship between serum LH level and endometrial thickness with endometrial hyperplasia. Besides, we want to compare serum LH levels in PCOS women with different BMI.

## Materials and Methods

In this cross-sectional study, three hundred fifty PCOS infertile women were enrolled between December 2009 and March 2011 in Royan Institute which is a referral-based fertility and endocrinology clinic. The present study was approved by the Institution Review Board and Ethics Committee of Royan Institute. The research was performed in accordance with Helsinki Declaration and acted in compliance with the committee of Publication Ethics (COPE) guidelines. All participants signed informed consent. The diagnosis of PCOS was based on the 2003 Rotterdam criteria (2). Cases with hyperprolactinemia, thyroid dysfunction, hypothalamic amenorrhea, Cushing’s syndrome and ovarian failure were diagnosed by hormonal investigations and excluded from this study. Eligible PCOS patients were asked about menstrual retardation, if the patient had had a menstrual retardation, beta-human chorionic gonadotropin (β-hCG) would have been checked, then in the absence of pregnancy, endometrial thickness was measured by trans-vaginal ultrasound, and endometrial biopsy was taken on the same day. If the patient had not had a delay in menstruation, endometrial thickness would have been measured and endometrial biopsy had been taken on the same day as well. Serum LH level was measured during the next cycle’s days 2 or 3 in patients with regular menstrual cycles and after administration of progesterone in patients with irregular menstrual cycles. Irregular menses defined as menstrual periods were shorter than 21 days or longer than 35 days. Intermenstrual interval was recorded and divided into two groups fewer than 3 months and 3 or more than 3 months. Endometrial thickness was measured by using trans-vaginal ultrasound by the same gynecologist for all patients. In the same way, an endometrial biopsy was taken by using a pipelle instrument (Endo cell, wallanch surgical devices Inc., orange, CT, USA) by the same gynecologist.

The endometrial tissues were sent for pathological diagnosis. All specimens were diagnosed by the same pathologist. The world health organization (WHO) criteria were used for the diagnosis of endometrial hyperplasia (15). Endometrial hyperplasia was reported as a morphologic classification into four classes of hyperplasia, composed of complex or simple architecture combined variously with the presence or absence of cytologic atypia (14).

Before the initiation of treatment cycle, height and weight were measured by well-trained nurse. BMI was calculated as body weight in Kg divided by the square of height in meters. To investigate the relationship between serum LH concentration and BMI, the patients were divided into three groups in accordance with their BMI: normal (20<BMI≤25), overweight (25<BMI<30) and obese (30≤BMI).

### Statistical analysis

The data was statistically analyzed by using SPSS software version 20. P<0.05 was considered as statistically significant level. T test and Mann- Whitney U test were used to compare means on the basis of the result of normality test. With regard to the results of the KolmogorovSmirnov Normality-test for endometrial thickness (Z=2.57, P=0.0001), we used non parametric Mann-Whitney U test to compare endometrial thickness between the two groups and the result revealed a significant difference among groups (Z=2.32, P=0.020). One way analysis of variance (ANOVA) was used to compare LH means between three BMI groups. Chi-square test was used to compare nominal variables between groups. We used multivariate logistic regression by backward to determine predictive factors for endometrial hyperplasia. Female age, BMI, serum LH level and endometrial thickness were included in the regression model.

## Results

Three hundred fifty infertile PCOS patients were involved in this study. The women’s age, BMI and duration of infertility were 28.5 ± 4.4 year, 28.8 ± 5.1 kg/m^2^ and 7.2 ± 4.4 year (mean ± SD) respectively. We found the frequency of endometrial hyperplasia was 2.6%. Basic characteristics of participants are summarized and illustrated in Table 1.

Table 2 shows participants’ endometrial pathology reports. Endometrial hyperplasia (simple, complex with or without atypia) was reported in 9 cases and a normal pathology (proliferative, secretory and polyp) was reported in 313 cases. Twenty eight biopsies were reported inadequate.

The mean of endometrial thickness in the normal group was 7.90 ± 2.86 mm and in the hyperplasic group was 10.78 ± 3.70.

Although other characteristics of two groups were not similar, no statistical significant difference was found between normal and hyperplastic groups (Table 1).

**Table 1:** 


		Normal n=313 Mean (SD)	Hyperplasia n=9 Mean (SD)	Total n=350 Mean (SD)	P value

Age		28.45 (4.42)	29.67 (4.74)	28.54 (4.41)	0.416**
Age of menarche		13.30 (1.69)	13.00 (1.32)	13.27 (1.65)		0.756***
Duration of infertility		7.15 (4.45)	8.94 (5.18)	7.21 (4.45)	0.279***	
LH level		8.41 (6.67)	9.47 ( 6.16)	8.42 (6.49)		0.600***
BMI		28.81 (5.11)	30.96 (6.08)	28.82 (5.14)	0.286***	
		n(%)	n(%)	n(%)		
Type of infertility	Primary	260 (83.1%)	8 (88.9%)	294 (84.0%)	0.537****
	Secondary	53 (16.9%)	1 (11.1%)	56 (16.0%)	
Menstrual Pattern	Regular	18 (5.3%)	0 (0%)	18 (5.1%)	0.592****
	Irregular	323 (94.7%)	9 (100%)	332 (94.9%)	
IMI	<3 month	162 (47.8%)	7 (77.8%)	169 (48.6%)	0.070****
	≥3 month	177 (52.2%)	2 (22.2%)	179 (51.4%)	


*; 28 (8.0%) of pathology reports were inadequate, **; t test, ***; Mann-Withney test, ****; Fisher exact test, IMI; Inter menstrual interval, LH; Luteinizing hormone, BMI; Boy mass index and PCOS; Polycystic ovary syndrome.

**Table 2:** Pathology report of endometrial biopsy in PCOS women


Report	n	% (valid)	Classification

Proliferative	216	61.6 (67.1)	Normal
Secretory	94	26.9 (29.2)	n=313, 89.4%
Polyp	3	0.9 (0.9)	
Simple hyperplasia	5	1.4 (1.6)	Hyperplasia
Complex hyperplasia with atypia	3	0.9 (0.9)	n=9, 2.6%
Complex hyperplasia without atypia	1	0.3 (0.3)	
Total	322	92.0 (100)	
Inadequate (missing)	28	8.0	


**Table 3:** Comparison of serum LH level and endometrial thickness in three groups (normal, overweight and obese) of PCOS women


BMI groups	Normala n=82	Overweightb n=148	Obesec n=120	P value

Serum basal LH	10.39** ± 7.4	7.89 ± 6.9	7.73 ± 4.8	0.007*
Endometrial thickness	7.9 ± 3.1	7.7 ± 2.7	8.0 ± 2.9	0.68


We also compared serum LH level and endometrial thickness in three groups (normal, overweight and obese) of participants. Results are illustrated in Table 3. One way ANOVA was performed which showed serum LH levels were not equal between groups (F=5.05, P=0.007). Least significant difference (LSD) post hoc test was also conducted which showed that the LH level in normal BMI group was significantly higher than that of other groups (P=0.005 and P=0.004), but there was no statistical difference between overweight and obese groups (P=0.841). There were no significant differences among three BMI groups in terms of endometrial thickness (P=0.6).

Multivariate logistic regression test demonstrated that the endometrial thickness was predictive factor for endometrial hyperplasia in PCOS women (odds ratio: 1.26, 95% confidence interval, 1.05-1.53, P=0.01). Female’s age, BMI and LH level weren’t predictive for endometrial hyperplasia.

## Discussion

Important risk factors for endometrial cancer in PCOS women were reported in previous studies including obesity, age≥50 years, nulliparity, hypertension, infertility and diabetes (7, 16-18). Therefore, PCOS women particularly those with chronic anovulation may be exposed to higher risk of endometrial hyperplasia and endometrial cancer. The mechanisms which cause endometrial hyperplasia and carcinoma are possibly hyperestrogenemia. Hyperandrogenism, hyperinsulinemia and obesity are also risk factors (19, 20). Hyperinsulinemia stimulates adrenal and ovarian androgen production, endogenous estrogen production from progesterone, and it also decreases hepatic sex hormone binding globulin production (18, 21). On the other hand, the combination of insulin resistance and hyperinsulinemia seems to increase the circulation of androgen levels (22, 23), and induce constant production of LH (24). Insulin, androgens and estrogens raise mitotic activity through insulin-like growth factor (7, 18). All These alterations motivate endometrial proliferation and mutagenic potential, which may elevate the risk of endometrial hyperplasia and ca ncer (18).

The prevalence of endometrial hyperplasia in our study was 2.6%. Holm et al. (18), in a large cohort of Danish premenopausal women (n=963) with PCOS found a low prevalence of endometrial thickness (1%) and endometrial cancer (0.1%). In comparison to previous study, our population had higher risk of hyperplasia; this difference may be related to variation of PCOS phenotypes between two different races.

In our survey, only endometrial thickness was predictive of hyperplasia. It means that for every 1 mm increase in endometrial stripe thickness, the odds ratio of hyperplasia increased by 1.26. Higher endometrial thickness in hyperplastic group in our study is similar to Cheung (10) and McCormick et al. (25) studies. They reported the only endometrial stripe thickness was predictive of hyperplasia and for every 1 mm increase in endometrial stripe thickness the risk of hyperplasia increased by 1.48. On the other hand, some previous studies had contrast results about the usefulness of endometrial stripe thickness in PCOS patients. We could not find statistical significant difference in serum LH level between normal and hyperplastic groups either. We could not find any other studies conducted on the relationship between serum LH level and hyperplasia. In our study, serum LH level was higher in hyperplastic group, but the difference was not statistically significant. Similar to the previous studies, we could not find any relationship between age and endometrial hyperplasia (10, 26).

As the menstrual cycle length increased and PCOS women extended menstrual cycles of more than 60 days, they were at risk of endometrial hyperplasia (27). In our study similar to McCormick et al. (25) inter menstrual interval was not associated with hyperplasia, but prior studies whose participants had longer durations of amenorrhea reported conflicting results (8, 26).

Similar to previous studies (11, 13), we observed serum LH Level to be significantly higher in some PCOS women with normal BMI, but Hendriks et al. (6) had found no correlation between LH concentration and age or BMI in PCOS patients. Pagán et al. (14) found the LH pulse frequency is elevated in PCOS, but no influence of BMI on either marker of hypothalamic function was detected. In PCOS, the pituitary response to a weightbased dose of GnRH is inversely related to BMI, these evidences suggested that in PCOS patients the effect of BMI on LH be interposed at a pituitary and not a hypothalamic level.

The present study reveals that there is no relationship between BMI and endometrial thickness in PCOS patients in compliance with to Iatrakis et al. (28) study. In contrast, McCormick et al. (25) reported that women with hyperplasia had significantly higher BMI in comparison with those without hyperplasia. Heller et al. (29) reported that higher BMI was associated with endometrial hyperplasia in comparison with lower BMI. Likewise, Zeng et al. (30) compared endometrial thickness and endometrial blood flow in three BMI groups in non-PCOS patients; they found no relationship between BMI and endometrial thickness in these patients. They also reported obesity (BMI≥28 kg/m^2^) seems to have a negative effect on endometrial and subendometrial blood flow. Due to the limitations, we did not evaluate endometrial pattern, endometrial spiral arterial resistance index (RI) and pulsatility index (PI) values and systolic/ diastolic ratio (S/D) in PCOS patients in our study. We suggest comparing these variables among normal weight, overweight and obese PCOS women in future studies.

## Conclusion

Sonographic endometrial stripe thickness is predictive for endometrial hyperplasia in PCOS women. We could not find any relationship between serum LH level and BMI with endometrial thickness in PCOS patients. However, our study confirmed a diverse relationship between serum LH level and BMI in PCOS patients.
